# ZnO:CuO Composites Obtained by Rapid Joule Heating for Photocatalysis

**DOI:** 10.3390/ma17143502

**Published:** 2024-07-15

**Authors:** Adrián Fernández-Calzado, Aarón Calvo-Villoslada, Paloma Fernández, Belén Sotillo

**Affiliations:** Materials Physics Department, Faculty of Physics, Complutense University of Madrid, 28040 Madrid, Spain; adriaf04@ucm.es (A.F.-C.); aaroncal@ucm.es (A.C.-V.); bsotillo@ucm.es (B.S.)

**Keywords:** zinc oxide, copper oxide, photocatalysis, Joule heating, galvanic corrosion

## Abstract

Semiconductor oxides belonging to various families are ideal candidates for application in photocatalytic processes. One of the challenges facing photocatalytic processes today is improving their efficiency under sunlight irradiation. In this study, the growth and characterization of semiconductor oxide nanostructures and composites based on the ZnO and CuO families are proposed. The selected growth method is the resistive heating of Zn and Cu wires to produce the corresponding oxides, combined with galvanic corrosion of Zn. An exhaustive characterization of the materials obtained has been carried out using techniques based on scanning electron microscopy and optical spectroscopies. The method we have followed and the conditions used in this study present promising results, not only from a degradation efficiency point of view but also because it is a cheap, easy, and fast growth method. These characteristics are essential in order to scale the process beyond the laboratory.

## 1. Introduction

Among the different types of materials, Transition Metal Oxides (TMOs) are gaining relevance every day due to their wide range of applications, versatility, and advantageous physical properties (conductive, magnetic, luminescent, etc.), enabling the fabrication of multifunctional systems [1]. However, for these applications to be really viable, much deeper knowledge about critical factors such as carrier concentration, recombination rates, mobility, defect structure, and their influence on the band structure (and hence on most relevant properties) is needed. Nanowires, and nanostructures in general, have been studied in recent decades for their different properties and applications compared to those of bulk materials and, in particular, for their increase in surface-to-volume ratio, which makes them much more efficient in many applications such as gas sensing [2,3,4,5] or photocatalysis [3,6,7,8,9,10,11]. Besides the physical properties required for an application, material selection criteria such as stability, low toxicity, non-criticality, low price, and versatility are of the utmost importance. On the other hand, the fabrication of hybrid or composite micro- and nanostructures is becoming a very active field [12,13,14,15,16,17,18], and the presence of several metal oxides in applications such as photocatalysis or sensing is attracting increasing interest [3,19,20].

A variety of methods have been used to grow these structures on various substrates or in different environments, with the aim of discovering new physics at the nanoscale. Our goal is to grow nanostructures from a metal oxide to increase the surface-to-volume ratio. Methods such as CVD (Chemical Vapor Deposition) or VLS (Vapor–Liquid–Solid) methods have typically been used for the growth of structures [6,21,22], but in most cases, they require long treatment times and can be expensive. A fast and cheap alternative is resistive heating that uses the Joule energy losses that appear in a metal when a current is circulated through it. In the presence of an ambient atmosphere, the hot metal wire oxidizes and a core (metal wire)/shell (oxide layer) is formed. Oxidation takes place in seconds; hence, this method allows us to greatly reduce treatment time and costs while achieving a high density of nanostructures [23,24,25].

As has been mentioned, the variety of possible applications of these nanostructures is very broad. In this work, we will focus on photocatalytic activity. Nowadays, catalytic processes are probably one of the hottest topics, since they are involved in environmental problems as severe as the production of hydrogen by water splitting and the elimination of pollutants from water. In water remediation processes, photocatalysis plays a central role [11], given the increasing problem of water contamination by effluents released from the rising expansion of textile, leather, paper, and ink industries. Heterogeneous photocatalysis based on TMOs is a process by which light absorption by the photocatalyst results in photoexcited electrons. These are transferred from the valence band (VB) to the conduction band (CB), resulting in electron–hole pairs. Such pairs are able to reduce and/or oxidize the molecules of the adsorbed compound (f.i. azo dyes) on the surface of the catalyst, degrading them to form CO_2_, H_2_O, and simple mineral acids [26,27,28,29].

**Figure 1:**
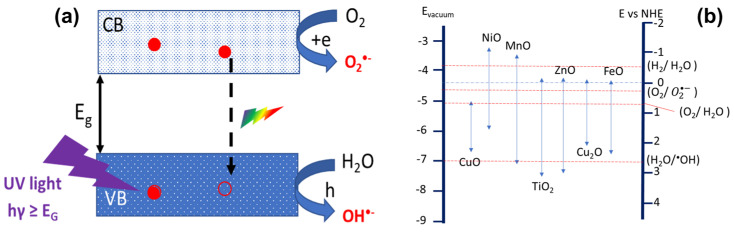
(**a**) A schematic illustration of the photocatalysis process in a TMO. (**b**) A schematic comparison between the bandgaps of some relevant TMOs and some common redox pairs.

In the case of TMOs, the catalytic activity originates basically from two sources: the generation of ^•^OH radicals through the oxidation of H_2_O, and the generation of O_2_^•−^ radicals through the reduction of O_2_. An electron can be transferred to an acceptor molecule if its reduction potential is below the CB of the photocatalyst. Correspondingly, a hole can be transferred to an acceptor molecule if its reduction potential is above the VB of the photocatalyst. The basic photocatalytic mechanism is schematized in Figure 1a. The pre-requisite for a TMO to be efficient as a REDOX mediator is that the redox potential for the radicals or reactants involved (for instance, in the case of photocatalysis, E_o_ (H_2_O/^•^OH) = 2.8 V and E_o_ (O_2_/$O_{2}^{•-}$) = 0.28 V vs. NHE) lies within the bandgap of the TMO (Figure 1b).

To date, different TMOs have been investigated for photocatalytic applications. The main characteristics of the material that determine its potential use as a photocatalyst are a suitable range of forbidden energies (bandgap), the lifetime of the photogenerated e^−^/h^+^ charge carriers, charge carrier transport to surface reaction sites, their morphology, a high surface-to-volume ratio, their stability, and their capacity to be reused (recovered from reactor). ZnO has been widely used as photocatalyst; however, due to the wide bandgap, its efficiency under sunlight is very limited, requiring the use of ultraviolet irradiation to trigger the photocatalytic process, although several strategies are used to overcome this limitation [22,30,31,32,33,34,35,36]. The two basic approaches are surface modification by sensitization or coupling with a narrow bandgap material, and bandgap engineering by creating defect states within the bandgap, for instance, by doping with transition metals of rare earth elements that might create levels in the bandgap associated to the d or f partially filled shells [22].

Another approach is the creation of hybrid structures by combining the active oxide with another material with a different bandgap, creating a heterojunction that not only modifies the light absorption properties but also prevents carrier recombination, enhancing the photocatalytic activity [27,35]. ZnO- and CuO-based systems are highly suitable for creating new architectures in combination with many other systems [1,37,38].

## 2. Materials and Methods

**Figure 2:**
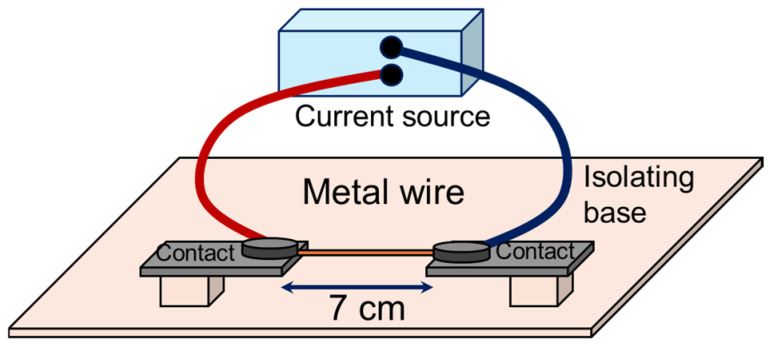
Experimental diagram of resistive heating system.

The precursor materials were Zn and Cu wires of 0.25 mm and 0.2 mm in diameter, with a purity of 99.99% and 99.9%, respectively. The resistive heating method used to carry out the heat treatments consisted of placing wires of about 7 cm in length with the ends contacted to metal electrodes, and then circulating a current through them. The experimental system used is shown in Figure 2. 

Using this procedure, three sets of samples have been prepared. Single metal wires of Cu and Zn were oxidized to form Zn/ZnO and Cu/CuO core–shell structures (series single wires, SWs). The second set was produced when each of the metal wires was oxidized in the presence of powder of the other element. Zn was oxidized while immersed in a CuO powder bath, and conversely, the Cu wire was oxidized while immersed in a ZnO powder bath (series immersed wires, IWs). This procedure has been previously reported as effective to obtain doped ZnO [23]. Finally, a series of braided wires, BWs, where the Cu and Zn wires were twisted together forming a sort of braid prior to the passage of the electric current, formed in this case not only Zn/ZnO and Cu/CuO but also CuO/ZnO heterojunctions. 

To characterize the morphology of the nanostructures, an FEI Inspect SEM scanning electron microscope was used (FEI Company, Eindhoven, The Netherlands). The compositional analysis of the samples was performed with X-ray microanalysis (EDS) with a Bruker detector, AXS Quantax, coupled to a Leica 440 SEM (Leica Cambridge Ltd., Cambridge, UK), or a QUANTAX 70 detector (Bruker, Berlin, Germany) incorporated into a Hitachi TM3000 (Hitachi High Technologies Corporation, Tokyo, Japan). The photoluminescence (PL) of the samples was analyzed using a LabRAM HR800 confocal microscope (Horiba JobinYvon, Villeneuve d’Ascq, France), excited with a 325 nm wavelength He–Cd UV laser and a 633 nm He–Ne laser for Raman spectroscopy. To investigate the cathodoluminescence (CL) of these samples using SEM, a Hamamatsu PMA-12 CCD (Shizuoka ken, Japan) was used on a Hitachi S-2500 SEM (Tokyo, Japan).

The photocatalysis experiments were performed using Rhodamine B as a pollutant simulator. A stock solution with 10 mg of the dye (Rhodamine B, Sigma-Aldrich (Saint Louis, MO, USA), with a purity greater than or equal to 95%) in 10 mL of distilled water was prepared. This resulted in a concentration of 100 ppm. Subsequently, to achieve the 2.5 ppm we used in the photocatalysis tests, 12.5 mL of stock solution was diluted in 500 mL of distilled water. We checked the degradation of the dye by measuring UV and visible light absorption curves with a Shimadzu UV-1603 spectrophotometer (Shimadzu Corporation, Kyoto, Japan) and with a UV-vis Jasco V770 (Jasco, Madrid, Spain) as a function of illumination time. The degradation experiments were performed by adding the Joule oxidized wires into 5 mL of dye solution and placing the glass containers into a homemade reactor. The illumination was performed using commercial LED strip lights (2 strips of 25 W for UV (peak wavelength at 405 and 365 nm) and 2 strips of 35 W (CRI > 90) for simulating sunlight) purchased from growthejungle.com (accessed on 1 June 2024).

## 3. Results

### 3.1. Characterization of the Samples

#### 3.1.1. Series SW (Single Wire)

For a single Zn wire (SWZN), we performed current tests in the range of 3.2–4.2 A, obtaining the best results in terms of the density of nanostructures with a current of 3.8 A. Although experiments with different rates of increasing current have been performed, the best results in terms of the reproducibility and homogeneity of the grown structures are obtained when the application of the current is stepwise [39,40]. All of the treatments for this series were carried out for 40 s.

**Figure 3:**
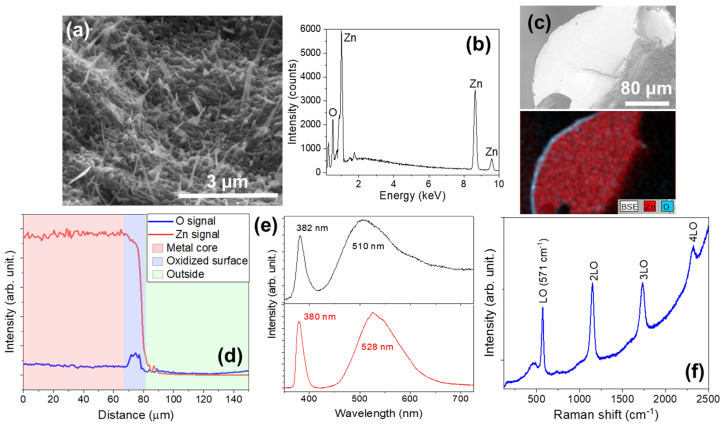
SWZN samples. (**a**) A SEM image of the ZnO nanowires obtained on the surface of the Zn wire. (**b**) The EDS spectrum recorded on the surface. (**c**) The BSE image and EDS map of the distribution of Zn (red) and oxygen (cyan) through the cross-section of the Zn wire. (**d**) The EDS signal profile along a line crossing the oxide layer. (**e**) CL (up) and PL (down) spectra. (**f**) The resonant Raman spectrum (λexc = 325 nm) obtained on the surface covered with nanowires.

After heat treatment, the sample SWZN shows a uniform layer of oxide coated with ZnO nanostructures. To study the morphology of these growths, we used scanning electron microscopy, as shown in Figure 3a. We observed a high density of homogeneously distributed nanowires, with lengths between 1 and 2 μm and diameters of up to a few hundred nanometers. 

As the heat treatment was performed in an ambient atmosphere, it was expected that oxidation would occur in the outer layer of the wire [23]. To confirm this fact, we performed EDS measurements (Figure 3b), which provided us with information about the elemental composition of the sample, in our case Zn and O. The results of the cross-sectional study show that the layer observed in the SEM (upper c) and EDS (lower c) images in Figure 3 is about 14 μm thick according to the measured EDS line (Figure 3d).

The formation of ZnO is confirmed by performing luminescence spectra. Figure 3e shows the PL and CL spectra, where we see the characteristic emission bands of ZnO. We identified the emission peak of the forbidden energy band of ZnO, and that was due to material defects [1,41,42,43,44]. Both PL and CL spectra show that the near-band edge (NBE) peaked at 380 nm without noticeable differences. On the contrary, visible differences are found between the PL and CL visible bands. In the first case, the band is considerably narrower and is centered at a slightly lower energy. Nevertheless, the relative intensities of the different components seem to be similar, giving a broad but almost symmetric band. The CL visible band is much broader and asymmetric, indicating the presence of several components with different relative intensities. The main peak is centered at 510 nm close to the high-energy component in the PL spectrum. The low-energy side of this band is not well defined, indicating the presence of several components of similar relative intensities extending toward the red part of the spectrum. A bump in this region is observed close to the lowest-energy component of the PL spectrum. These differences are attributed to the differences in the excitation source, since in the case of CL, not only can carriers be promoted to higher energy levels but a huge number of electrons that can populate the defect levels in the gap are introduced. 

To further assess the good crystallinity of the grown structures, Raman spectroscopy experiments were performed. The characteristic spectrum obtained by excitation with a 325 nm laser is shown in Figure 3c. Here, we can identify the characteristic peak of ZnO, corresponding to the E_2_^high^ (LO) mode which peaked at 571 cm^−1^ and its replicas, in agreement with previous works [45].

**Figure 4:**
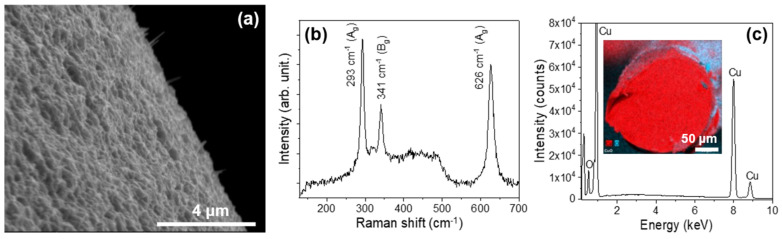
SWZN samples. (**a**) A SEM image of the CuO nanowires and microcrystals obtained on the surface of the Cu wire. (**b**) The Raman spectrum (λexc = 633 nm) obtained on the surface covered with nanowires. (**c**) The EDS spectrum recorded on the surface. In the inset, EDS map of the distribution of Cu (red) and oxygen (cyan) through the cross-section of the Cu wire is presented.

On the other hand, the best condition for the growth of copper oxide on a Cu wire is a current of 7 A applied for 60 s. After the Joule heating process, Cu wires (SWCUs) show a coating made up of submicron crystals and nanowires of lengths of 1–2 μm. We have observed these structures using SEM, as shown in Figure 4a. As in the case of the zinc wire, the presence of the copper oxide layer has been assessed by Raman measurements (Figure 4b). As expected, the luminescent emission of CuO is very weak; therefore, the results of Raman spectroscopy are more reliable. In Figure 4b, the peaks corresponding to the active Raman modes in CuO are shown. The peak of 293 cm^−1^ corresponds to the A_g_ mode and the peaks of 331 and 626 cm^−1^ to the two B_g_ modes [46,47].

The composition of the wires was studied by EDS (Figure 4c), and the peaks observed confirm the presence of Cu and O in the crust. The cross-section allowed us to estimate the layer thickness, about 6 μm (Figure 4c).

#### 3.1.2. Series IW (Immersed Wires)

In order to prepare the first set of composite samples, the wire of one of the metals was immersed in oxide powder of the other metal (i.e., Zn wire in CuO and Cu wire in ZnO). The required currents to achieve growth are higher than for the SW series, since a larger amount of mass has to be heated (wire + powder). For the Zn wire in CuO (IWZN), the optimal current is 4.2 A, applied for 30 s. It is observed that higher currents or larger periods of time lead to the failure of the Zn wire at the region that is outside the powder.

**Figure 5:**
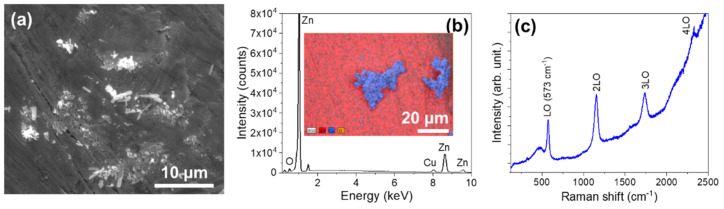
IWZN samples. (**a**) A SEM image of the surface of the Zn wire immersed in CuO powder after Joule heating. (**b**) EDS measurements on the IWZN samples. The inset shows the compositional map, where the Zn signal is presented in red and the Cu signal in blue. (**c**) Raman spectra (λexc = 325 nm) of the surface of the IWZN wires.

The results obtained for the best IWZN sample are shown in Figure 5. SEM images show the formation of an oxide layer in which powder microparticles are embedded (Figure 5a). The density of nanowires is reduced in comparison with the SWZN samples, and the lengths are shorter (in the range of tens to hundreds of nm). X-ray microanalysis measurements indicate that the embedded powder is mainly copper oxide (Figure 5b), which is surrounded by a layer of ZnO, as confirmed by EDX and Raman (Figure 5c) measurements.

**Figure 6:**
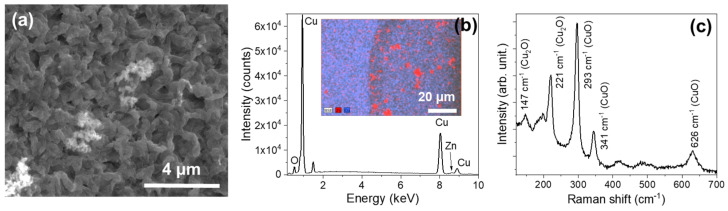
IWCU samples. (**a**) A SEM image of the surface of the Cu wire immersed in ZnO powder after Joule heating. (**b**) EDS measurements on the IWCU samples. The inset shows the compositional map, where the Zn signal is presented in red and the Cu signal in blue. (**c**) The Raman spectrum (λexc = 633 nm) of the surface of the IWCU wires.

On the other hand, for the Cu wire in ZnO powder, a current of 10 A for 120 s can be maintained without the failure of the Cu wire. This allows the wire to produce a thicker oxide layer with microcrystals, as shown in Figure 6a. EDX measurements confirm that zinc oxide powders are embedded in the matrix of copper oxide microcrystals (Figure 6b). More information is obtained from the Raman spectra performed (Figure 6c). Peaks associated with CuO modes are located at 293, 341, and 626 cm^−1^, similar to those observed in the SWCU samples. However, new peaks are observed in this case, at 147 and 221 cm^−1^. They can be ascribed to Cu_2_O [47] (T_1u_ and 2E_u_, respectively). The appearance of this lower oxidation number oxide may be related to the growth conditions, as the wire is in contact with ZnO powder instead of with the air, reducing the amount of oxygen available in the process. This effect can also be related to the reduced density of nanowires obtained in the IWZN samples.

#### 3.1.3. Series BWs (Braided Wires)

**Figure 7:**
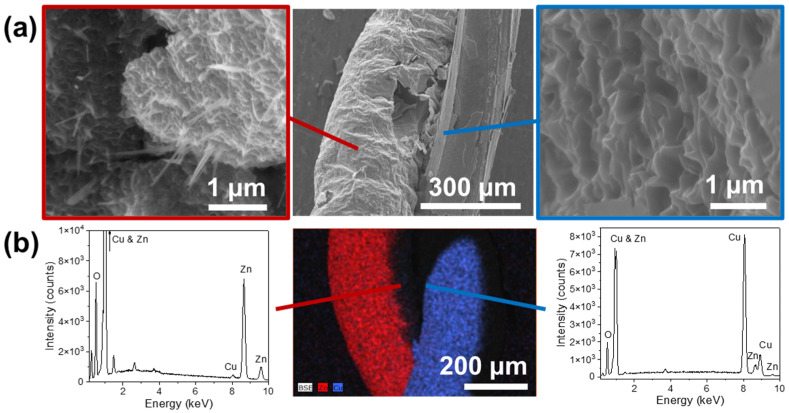
(**a**) A SEM image of the braided nanowires: Cu wire (right) and Zn wire (left). (**b**) X-ray maps (center) and spectra (Cu wire (right) and Zn wire (left)).

Finally, a third series was prepared by twisting together two wires, one of each element. A more complex structure is then obtained, since both oxide layers might show cross-doping (ZnO:Cu and CuO:Zn) to some extent. Besides cross-doping effects at the closest contact points, ZnO:CuO heterojunctions might form. The Cu and Zn wires were strongly crisscrossed and fed into the Joule heating system. In this case, it was determined that the optimal growing conditions were a 9A current for 60 s. With SEM, we saw a substantial improvement in the density of nanowires grown after heat treatment (Figure 7). EDS analysis of these samples shows effective cross-doping in both oxide shells (Figure 7b). We have confirmed the presence of copper in the Zn wire and vice versa. In addition, we see characteristic peaks of C and Al due to the specimen holder and the carbon tape used to fix the samples to the sample holder.

### 3.2. Photocatalysis Experiments

Photocatalysis experiments have been performed with all three series. As mentioned in the Section 2, Rhodamine B was used to simulate the pollutant. The degradation process was monitored by recording the UV-Vis absorption spectra of the solution with the different catalysts as a function of illumination time [48,49], and the degradation efficiency was calculated using the following expression:

$$\%=\frac{C_{0}-C_{t}}{C_{0}}\times 100\%$$

where C_0_ is the initial concentration and C_t_ is the concentration at time t, both computed from the maximum of the Rhodamine absorption spectrum (at 554 nm). The degradation efficiency was determined for all of the samples after 6 h of UV plus visible light illumination, and both UV and visible illumination separately. This irradiation time is longer than in other reports due to the lower power of the lamps and the smaller concentration of the catalyst. That is, the catalyst is not the whole wire but just the micrometric oxide coating.

**Figure 8:**
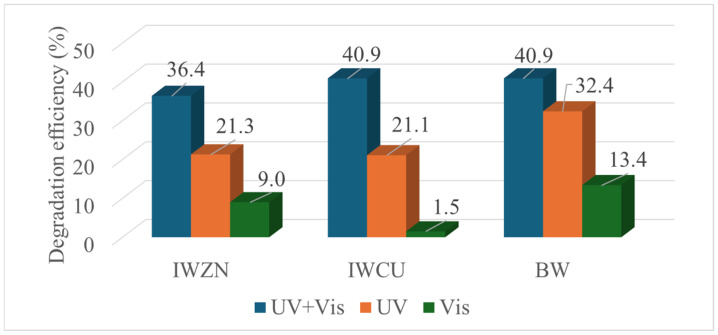
Photocatalytic performance of different sets of samples.

The wires from the SW series show poor degradation rates under UV+Vis illumination, obtaining a degradation efficiency of 12% for the SWZN samples and of 1.5% for the SWCu samples after 6 h of irradiation. Better results are observed for the sample sets with ZnO/CuO composites. The results for all of the illumination conditions are shown in Figure 8.

**Figure 9:**
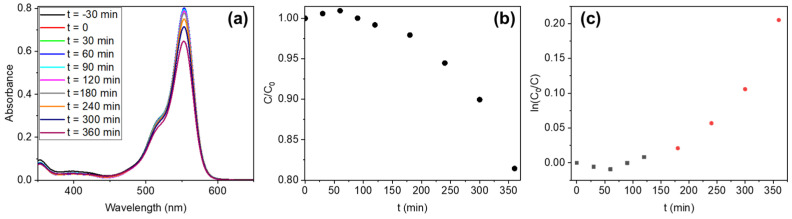
(**a**) Rhodamine UV-Vis absorption spectra variation over time with BWs as photocatalysts and UV+Vis illumination. (**b**) C/C_0_ variation over time for BW samples. (**c**) Pseudo-first-order kinetics for Rhodamine dye in the presence of BWs as photocatalysts. Black squares and red circles indicated the two linear behaviors observed.

From the graph in Figure 8, it is clearly observed that the samples with the better performance are those of the BW series. This is particularly true for the visible illumination conditions, although the efficiency is considerably improved when the combination of both UV and visible light is used. Since one of our main goals is to study the materials appropriate for solar photocatalysis, this series was selected for the detailed kinetic study. The Rhodamine absorbance and the concentration ratio is plotted as a function of time (Figure 9a,b), for a 100 mL solution of 2.5 ppm of Rhodamine B with five BWs inside it. We have observed that the degradation is negligible for the first 3 h, whereas it starts to be more effective after that period. Two regimes can be identified when plotting the pseudo-first-order kinetics (ln (C_0_/C) versus time, Figure 9c), the first with a rate constant of 9.2 × 10^−5^ min^−1^ and the second with a rate constant of 1.4 × 10^−3^ min^−1^.

**Figure 10:**
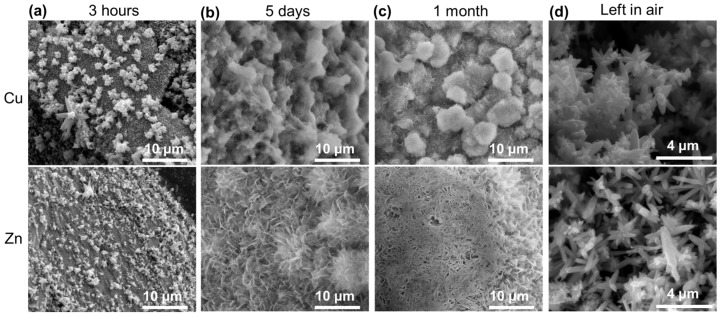
SEM images of the surface of the BW (Cu wire up and Zn wire down) after (**a**) 3 h, (**b**) 5 days, and (**c**) 1 month in the solution. (**d**) SEM images of the surface of the BW (Cu wire up and Zn wire down) once the sample has been left in air.

**Figure 11:**
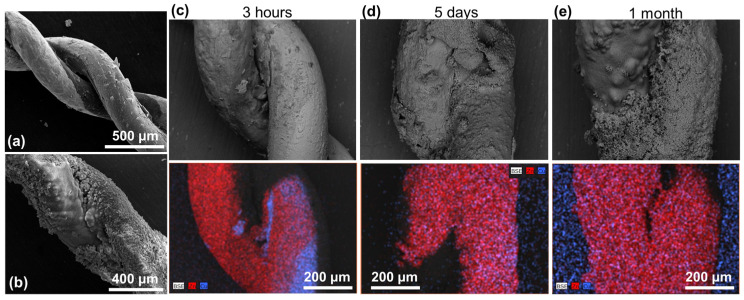
SEM images of the surface of the BW after (**a**) 3 h and (**b**) 1 month in the solution. EDS maps for the BW after (**c**) 3 h, (**d**) 5 days, and (**e**) 1 month in the solution. In the EDS maps, the Zn signal is presented in red and the Cu signal in blue.

To understand what is happening, the sample was collected and characterized after being immersed for 3 h in the solution. The SEM images are presented in Figure 10a for both Cu (up) and Zn wires. The morphology of the surface is completely different compared with the images in Figure 7a. On both wires, flower-like microstructures appear. Leaving the BW in the solution for longer periods of time (from days, Figure 10b, to a month, Figure 10c) shows that the modification of the sample continues, revealing flake-like microstructures. More interestingly, when the sample was left in air (Figure 10d), flower-like structures with hexagonal rods substituted the flakes. A comparison of the BW sample before (Figure 7a), after 3 h (Figure 11a), and after the whole process (Figure 11b) indicates that the sample surface is completely changed.

Further information about the modification that the sample is suffering is sought through EDS measurements (Figure 11c–e). If we compare the EDS maps with that presented in Figure 7b, it can be observed that Zn (red) starts to cover the Cu (cyan) wire after 3 h in the solution. The covering is almost completed after 5 days. After 1 month, it is not possible to identify the Cu wire due to the thickness of the Zn cover. From these observations and considering that Zn-Cu forms an efficient galvanic pair, the phenomenon may be related to galvanic corrosion of Zn. This effect has been previously reported by Takahashi et al. in Zn and Cu plates put in contact in neutral water under UV irradiation [50]. The formation of ZnO on Cu through galvanic corrosion has been reported to increase the visible photocatalytic performance [51,52], in agreement with our results.

**Figure 12:**
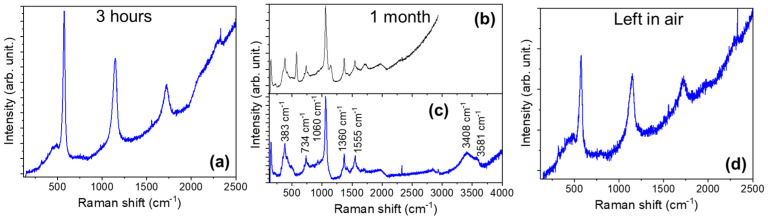
Raman spectra (λexc = 325 nm) recorded on the surface of the BW samples after (**a**) 3 h and (**b**,**c**) 1 month in the solution. (**d**) Raman spectra (λexc = 325 nm) when the sample is left in air for several days after galvanic corrosion.

Raman measurements have been performed to corroborate the galvanic corrosion. The spectra are gathered in Figure 12. When the process has been carried out for 3 h, flower-like structures made of hexagonal microrods present the characteristic resonant Raman modes of ZnO (Figure 12a). When the process is extended for more days (until 1 month), two different Raman spectra are detected (Figure 12b,c). The peaks at 383, 734, 1060, 3408, and 3581 cm^−1^ are ascribed to a zinc hydroxide, with the last two peaks related to -OH vibrations (Figure 12c). Peaks at 1360 cm^−1^ and 1555 cm^−1^ are related to carbonate vibrations; so, all of these modes indicate the formation of zinc hydroxide carbonate or hydrozincite (Zn_5_(OH)_6_(CO_3_)_2_). The formation of this compound has been observed in the corrosion of Zn in marine environments [53].

Combining the observations by Takahashi et al. and Li et al. [50,53], the reaction that is produced is as follows: First, the galvanic reaction is established when Zn and Cu are in contact inside the solution, releasing Zn^2+^, e^−^, and OH^−^. As there is no Cu^2+^ in the solution, zinc loses electrons and migrates toward regions where OH^−^ is accumulated (mainly in the vicinity of the Cu surface) and reacts to form zinc hydroxide carbonate:

$$5Zn^{2+}++6OH^{-}+2CO_{3}^{2-}\;\to \;Zn_{5}{\left(OH\right)}_{6}{\left(CO_{3}\right)}_{2}$$

where CO_3_^2−^ comes from the degradation products of Rhodamine B. Once zinc hydroxide is deposited, it is further decomposed into ZnO, as confirmed by the appearance of ZnO resonant Raman modes (Figure 12b). If the sample is left in air, galvanic corrosion is stopped, and all of the zinc hydroxide is finally transformed into ZnO (Figure 12d). According to Takahashi et al. [50], the presence of ZnO nanoparticles promotes the enhancement of the local alkaline region through photochemical reactions; so, the presence of the ZnO nanowires formed by Joule heating is assisting the galvanic process:

$$ZnO+h\nu \to ZnO\;\left(e^{-}+h^{+}\right)$$

$$H_{2}O+h^{+}\to •OH+H^{+}$$

$$•OH+e_{aq}^{-}\to OH^{-}$$

Finally, we tested the degradation performance of the BW once it was covered completely in ZnO rods produced by galvanic corrosion (Figure 10d). After 6 h of UV+Vis illumination, a degradation efficiency of 23% is determined, whereas an efficiency of 25% and 8% is obtained under UV and visible irradiation, respectively. If we compare these values with those for the BW fresh samples in Figure 8, it can be inferred that the exchange between Zn and Cu during galvanic corrosion is helping in the photocatalysis process.

## 4. Conclusions

All of the sample series obtained by Joule heating have a uniform layer of metal oxide grown in very short treatment times, in the order of tens of seconds. The efficiency of this method is indisputable compared to other more expensive and time-consuming options. An additional advantage of this type of growth for the application we have selected, photocatalysis, compared to the use of suspended nanoparticles, for example, is its easy recovery. It is not necessary to subject the treated liquid to any subsequent process to collect the catalyst, as the wires are easily salvageable. This allows for the characterization of the photocatalyst after the process.

After finding the optimal growth conditions, the properties exhibited by ZnO/CuO composite samples are interesting for photocatalysis. The configuration that yields the best results is that of the Zn-Cu braided wires, due to their significant and uniform nanostructured coating, as well as slight mixed doping at the interface between the oxide layers. The photocatalysis tests performed, using Rhodamine B as a simulated pollutant, indicate that these samples produce significant degradation with both UV and visible illumination. The illumination of the contacted Zn-Cu braided wires, combined with the oxides formed on their surface by Joule heating, promotes the enhancement of the galvanic corrosion that produces ZnO nanorods, which help in the overall photocatalytic process.

## Data Availability

The original contributions presented in the study are included in the article, further inquiries can be directed to the corresponding author.
